# Single-Cell Electric Lysis on an Electroosmotic-Driven Microfluidic Chip with Arrays of Microwells

**DOI:** 10.3390/s120606967

**Published:** 2012-05-25

**Authors:** Chun-Ping Jen, Tamara G. Amstislavskaya, Ya-Hui Liu, Ju-Hsiu Hsiao, Yu-Hung Chen

**Affiliations:** 1 Department of Mechanical Engineering, Advanced Institute of Manufacturing with High-Tech Innovation, National Chung Cheng University, Chia Yi, 62102, Taiwan; E-Mails: black_12_28@hotmail.com (Y.-H.L.); ggina03@yahoo.com.tw (J.-H.H.); 2 Institute of Cytology and Genetics, Siberian Branch of Russian Academy of Sciences, Novosibirsk, 630090, Russia; E-Mail: amst@bionet.nsc.ru; 3 Department of Biochemistry and Molecular Biology, National Cheng-Kung University, Tainan, 70101, Taiwan

**Keywords:** microwell, single-cell, electric lysis, electroosmotic-driven, microfluidics

## Abstract

Accurate analysis at the single-cell level has become a highly attractive tool for investigating cellular content. An electroosmotic-driven microfluidic chip with arrays of 30-μm-diameter microwells was developed for single-cell electric lysis in the present study. The cellular occupancy in the microwells when the applied voltage was 5 V (82.4%) was slightly higher than that at an applied voltage of 10 V (81.8%). When the applied voltage was increased to 15 V, the cellular occupancy in the microwells dropped to 64.3%. More than 50% of the occupied microwells contain individual cells. The results of electric lysis experiments at the single-cell level indicate that the cells were gradually lysed as the DC voltage of 30 V was applied; the cell was fully lysed after 25 s. Single-cell electric lysis was demonstrated in the proposed microfluidic chip, which is suitable for high-throughput cell lysis.

## 1. Introduction

Conventional biochemical assays are performed using populations of cells to determine their quantitative biomolecular profiles. However, population averages do not reflect actual physiological processes in individual cells, which occur either on very short time scales (e.g., kinase signaling cascades) or nonsynchronously (e.g., response to an external chemical gradient) [1]. Biomedical devices created using microfabrication technologies allow the precise manipulation of biological cells, and thus have the potential to provide individual characterization, detection, and assay of cells at the single-cell level. Therefore, accurate analysis at the single-cell level has become a highly attractive tool for investigating cellular content. Microfabrication technologies combined with surface chemistry have stimulated research to understand the fundamental cell biology and pharmaceutical analysis by exposure of cells to drugs and environmental perturbations [2]. Many methods, such as microcontact printing, microfluidic patterning, and photolithography, have been employed to create micropatterned surfaces containing adhesive and non-adhesive regions for cells [3–6]. These approaches are limited to adherent cells and additional surface chemistry procedures are often required. Alternative methods that do not require adherent cells, including dielectrophoresis [7], optical tweezers [8] and selective dewetting [9], have been adopted for trapping single cells. However, these methods are not suitable for high-throughput applications. The approach of passively confining cells inside microwells has been proposed because of its simplicity and ease of operation. Park *et al.* [10] proposed a flow method that enables single-cell trapping in microwells with a size sufficient to allow attachment and division of captured cells. A simple method for trapping single cells in large open-top microwell arrays was developed and optimized by Rettig and Folch [11]. The parameters that maximize single-cell occupancy for two cell types, including the microwell diameter, microwell depth, and settling time, were also investigated in their study. Cell lysis is crucial in the analysis of intracellular components containing information about genetic or disease characteristics in genomics, proteomics, and metabolomics [12]. Cell lysis could be achieved by various approaches [13], such as optical [14], mechanical [15], chemical [16], and electric [17] methods. A method that combines optical trapping and microfluidic-based droplet generation for encapsulating single cells within a picolitre-size aqueous droplet was proposed by He *et al.* [18]. The trapped cells are lysed rapidly using a YAG laser with a 5-ns pulse duration. Continuous analysis of two dyes loaded into single mammalian cells using laser-based lysis combined with the electrophoretic separation of cell content was achieved using microfluidic chips [19]. The cells are mechanically lysed owing to a cavitation bubble generated by a single laser pulse from a 532-nm picosecond pulsed laser. Laser-mediated lysis is well suited for integration into microfluidic chip platforms; however, it requires complex experimental setups. In chemical lysis, a detergent is usually introduced into a cell membrane to create pores within the membrane and lyse the cell. Detergent lysis is well established for bulk biochemical assays. Single-cell capture by a dam-like structure and chemical lysis inside a closed volume was demonstrated in a microfabricated device [16]. Following cell lysis, a limited and stable dilution of intracellular components is used to simplify the requirements for downstream assays. Huang *et al.* [20] designed a microfluidic device to trap cells using a pair of valves. Then, the chamber, where the cell is immobilized, is filled with lysis buffer containing fluorescent antibodies for labeling proteins to quantify the protein contents of a single cell using single-molecule fluorescence counting. Adherent cells were analyzed serially using detergent lysis followed by capillary electrophoresis [21]. Electrophoretic buffer containing sodium dodecyl sulfate (SDS), which is a strong ionic detergent that achieves cell lysis on the order of seconds, was then introduced using sheath flow around the capillary inlet. A simple single-cell lysis method that uses a dense array of microwells (10–30 pL in volume) fabricated from poly(dimethylsiloxane) (PDMS) and a commercially available cell lysis reagent was developed [22]. After the cell lysis solution diffuses into a microwell from the flow cell, which is between a bottom coverslip and a top cell-trapping PDMS sheet separated by two strips of double-faced adhesive tape, the PDMS sheet is rapidly pressed against the bottom coverslip to close each microwell, thereby causing gradual cell lysis. McClain *et al.* [17] developed an integrated microfluidic device that automatically transports cells to an electrical lysis location. The cell lysate is injected into a separation channel and the labeled lysate contents are electrophoretically separated prior to laser-induced fluorescence (LIF) detection. A microfluidic device with different field strengths in geometrically defined sections of a microchannel was proposed to analyze intracellular contents from single cells after electrical lysis [23]. The electrophoresis of calcein AM (a fluorogenic dye) which was loaded in the cytoplasm prior to electric lysis was demonstrated the feasibility of analyzing cell lysate from single cells in their device. Furthermore, microfluidic devices with different geometries providing pulse-like electric field variation were applied to gene delivery by electroporation [24]. A continuous electrical cell lysis device in which the width and length of the microchannel changes to generate a focused high electric field strength for cell lysis was proposed [25]. A low electric field strength for the transport of samples at a low operational voltage is employed. The device generates a high electric field strength of 1.2 kV/cm at the orifice to disrupt red blood cells with a 100% lysis rate under an operational voltage of 50 V. A device capable of electrical cell lysis and the evaluation of lysis efficiency in continuous flow using dielectrophoretic cell sorting was proposed by Mernier *et al.* [26]. An AC electrical field is used at a frequency that optimizes cell lysis while avoiding the creation of bubbles at the electrode surface; the AC field causes a dielectrophoretic effect on the cells that can be used to increase the transit time of the cell in the lysis region. In the present study, a microfluidic chip with microwells of 30 μm in diameter, is developed for cellular patterning using a electroosmotic driven flow and the feasibility of electric lysis for human carcinoma cells (HeLa cells) at the single-cell level is demonstrated.

## 2. Materials and Methods

### 2.1. Fabrication of Microfluidic Chips with Microwells

**Figure 1.**
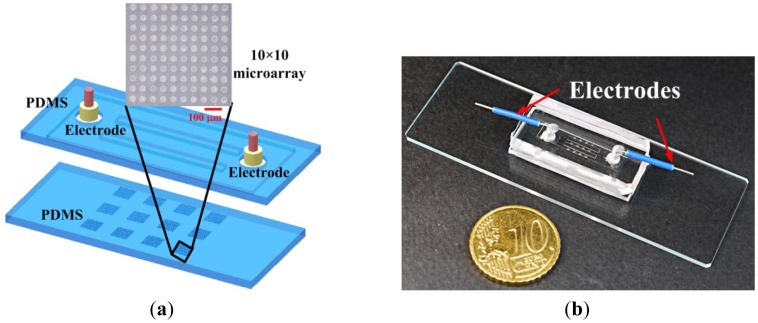
(**a**) Schematic diagram of the proposed microfluidic chip for single-cell-based microarrays; (**b**) Photograph of the completed microfluidic chip.

A biocompatible material, poly(dimethylsiloxane) (PDMS), was adopted for single-cell-based arrays in the microfluidic chip, as illustrated in Figure 1(a). The main channel, formed on the top PDMS layer, is 4.4 mm wide, 100 μm in height and 21 mm long. This main channel is divided into three microchannels, each 800 μm wide and 8 mm long, at the center region. Each microchannel contains four 10 × 10 microwells with 30 μm in diameter and 20 μm deep, on the bottom PDMS layer. The mold masters were fabricated by spinning SU-8 (SU-8 50, MicroChem Corp., Newton, MA, USA) on a silicon wafer to define the microwells and microchannel, respectively. The mold master of the microfluidic channels (around 100 μm in height) was fabricated by spinning SU-8 at 500 rpm for 20 s and then at 1,000 rpm for 35 s on the silicon wafer. The resist was soft baked on a hotplate at 65 °C for 10 min and then at 95 °C for 30 min. The resist was then allowed to cool to room temperature. The SU-8 was exposed to ultraviolet (UV) radiation at a dose of 200 mJ/cm^2^. The post-exposure baking was done at 65 °C for 3 min and then at 95 °C for 10 min. The exposed samples were developed with SU-8 developer for 5 min. The mold master of the microwells (around 20 μm in height) was fabricated by spinning SU-8 at 500 rpm for 20 s and then at 4,500 rpm for 35 s on a silicon wafer. The resist was developed with SU-8 developer for about 2 min after baking and exposure to UV radiation under the conditions mentioned above. PDMS prepolymer mixture (Sylgard-184 Silicone Elastomer Kit, Dow Corning, Midland, MI, USA) was poured and cured on the mold masters to replicate the patterned structures. After peeling off the PDMS replica with the microchannel, the inlet and outlet ports were made by a puncher. The two PDMS replicas were bonded after treatment with oxygen plasma in an O_2_ plasma cleaner (model PDC-32G, Harrick Plasma Corp., Ithaca, NY, USA). Electrodes were inserted into the inlet and outlet for applying the voltages. The distance between two electrodes is 15 mm. A photograph of the completed microfluidic chip is shown in Figure 1(b).

### 2.2. Cell Treatment

A human cervical carcinoma cell line (HeLa cells) was cultured for an experimental demonstration of single-cell lysis using the proposed microfluidic chips with microwells. The cells were serially passaged as monolayer cultures in DMEM medium (Gibco, Grand Island, NY, USA), with 3.7 g of NaHCO_3_ per liter of medium added, supplemented with 10% fetal bovine serum (FBS, Gibco) and 1% penicillin/streptomycin (Gibco). The cell culture dish (Falcon, Franklin Lakes, NJ, USA) was incubated in a humidified atmosphere containing 5% carbon dioxide at 37 °C; the medium was replaced every 1 to 2 days. Cells grown to sub-confluence were washed with phosphate-buffered saline (PBS, Biochrome, pH 7.4) and harvested by a 5-minute treatment with 0.25% trypsin and 0.02% ethylene diamine tetraacetic acid (EDTA, Sigma, St. Louis, MO, USA). The cells were stained using a standard fluorescence assay with calcein AM (Molecular Probes, Eugene, OR, USA) prior to the experiment. Calcein AM is a green fluorescent dye which is able to penetrate the cell membrane into the cytosol and transform into a fluorescent form when it is hydrolyzed by esterases located inside cells. The cell samples were then suspended in an 8.62 wt% sucrose solution to increase the osmolarity to normal physiological levels.

### 2.3. Experimental Procedure

**Figure 2.**
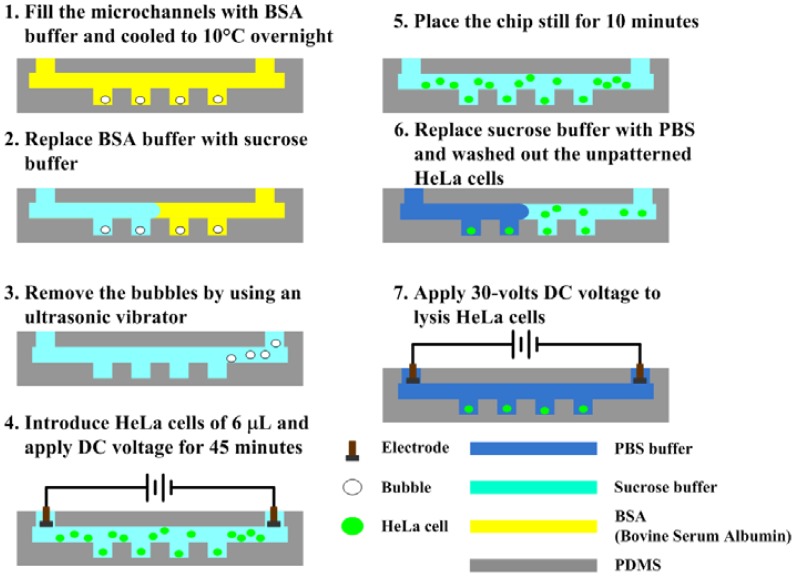
Experimental procedures for cell patterning.

The experimental procedures are illustrated in Figure 2. First of all, the microfluidic channel was washed and blocked overnight with bovine serum albumin (BSA) buffer (10 mg/mL) to avoid nonspecific absorption of cells. The sucrose solution was injected manually to replace the blocking buffer prior to experiments. The trapped bubbles within the microwells were removed using an ultrasonic vibrator. The cell sample of 6 μL, which has a concentration of 10^7^ cells/mL was dropped, and then a direct current (DC) voltage of 5, 10 and 15 V was applied for 45 min to electroosmotically drive the cells into the microwells. The sucrose solution was used herein as a working fluid for electroosmosis to avoid the occurrences of joule heating and bubble formation due to the medium with a high conductivity, for example the phosphate saline buffer (PBS) [27]. The microfluidic channel was washed with PBS buffer after that the chip was placed still for 10 min. Finally, a DC voltage of 30 V was applied to perform the electric lysis in the PBS buffer with a high conductivity, in which the required voltage for lysis could be reduced. The cellular deposition in microwells and the electric lysis were then observed and recorded by an inverted fluorescence microscope (model CKX41, Olympus, Tokyo, Japan) mounted on a CCD camera (DP71, Olympus, Tokyo, Japan) and controlled by a computer with Olympus DP controller image software. The fluorescent images were quantitatively analyzed using NIH ImageJ (National Institute of Health, Bethesda, MD, USA) to measure the intensity of fluorescence. The fluorescence intensity from each pixel can be analyzed with the ImageJ program.

## 3. Results and Discussion

**Figure 3.**
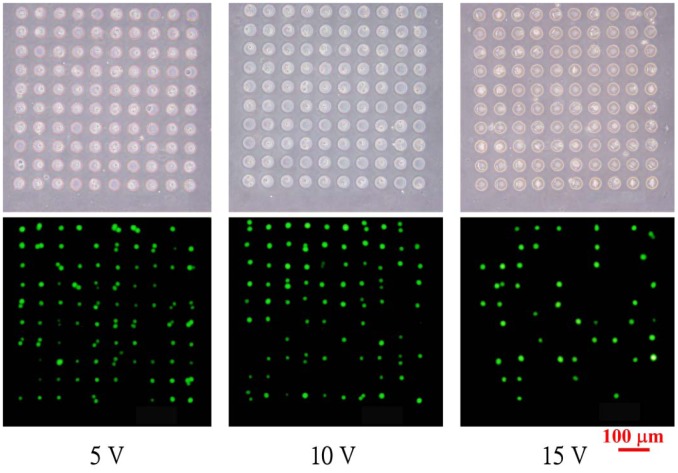
Bright and fluorescence images of micropatterned HeLa cells in microwells with diameters of 30 μm under different applied voltage for electroosmotic flow.

Micropatterned HeLa cells in the microfluidic chips with 30-μm-diameter microwells are shown in Figure 3; the applied voltages for electroosmotic flow are 5, 10 and 15 V, respectively. The occupancy of cells in the microwells when the voltages of 5 or 10 V are applied is higher than that for the applied voltage is 15 V. The fluorescent images of cells stained by calcein AM demonstrate the viability of the cells.

**Figure 4.**
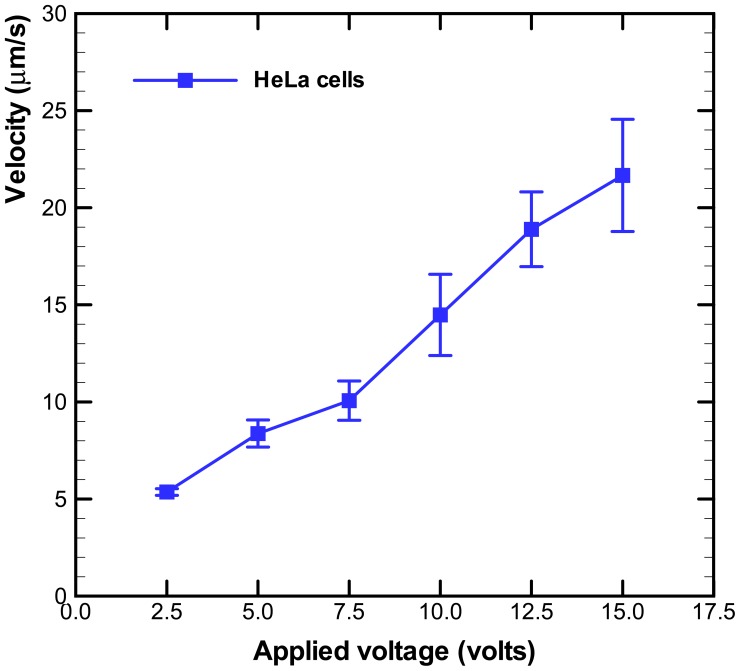
The electroosmotic-driven velocity of HeLa cells for different applied voltages.

**Figure 5.**
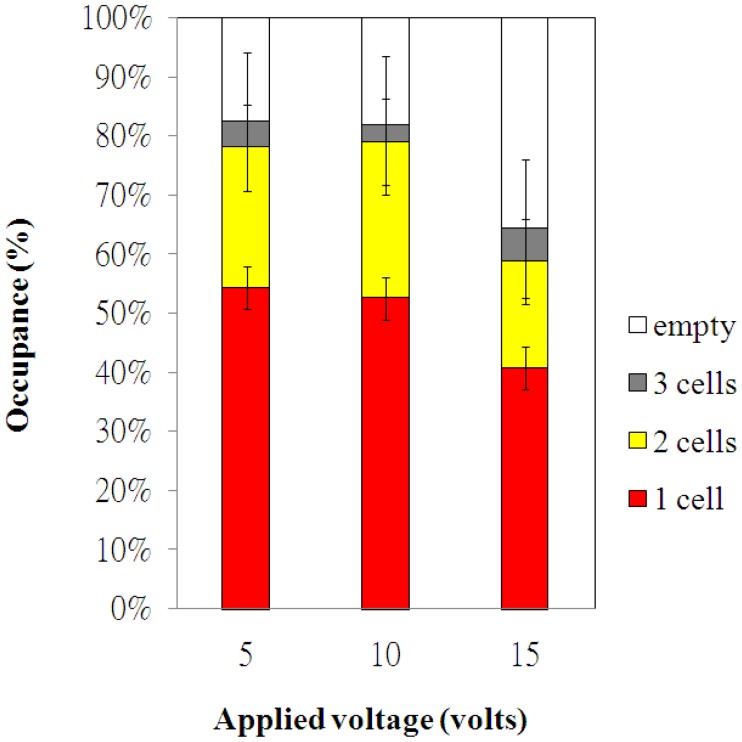
Distributions of 30-μm-diameter microwell occupancies for HeLa cells under different applied voltage for electroosmotic flow. The experimental data are based on manual counts of cells in twelve arrays of 10 × 10 microwells. Each experimental data point represents the average value and the error bar shows the standard error of the mean.

The electroosmotic-driven velocity of HeLa cells for different applied voltages are measured and plotted in Figure 4. The time of HeLa cells migrating a fixed distance (50 μm) was measured via the microscope. The measurements were repeated for more than three times for various applied voltages. The velocity of HeLa cells increased with the applied voltages, as shown in Figure 4. The experimental data of cell occupancy for HeLa cells in the microwells for various applied voltages are revealed in Figure 5. The experimental data is based on manual counts of cells in twelve arrays of 10 × 10 microwells using the inverted fluorescence microscope. Each experimental data point represents the average value, and the error bar shows the standard error of the mean. The occupancy of cells in the microwells decreases with increasing applied voltage due to the increasing velocity of HeLa cells. The cellular occupancy in the microwells when the applied voltage was 5 V (82.4%) was slightly higher than that in that (81.8%) at applied voltage of 10 V. When the applied voltage was increased to 15 V, the cellular occupancy in the microwells was dropped to 64.3%. More than 50% of the occupied microwells contain individual cells, as shown in Figure 5. Some of the 30-μm-diameter microwells contain two or three cells.

**Figure 6.**
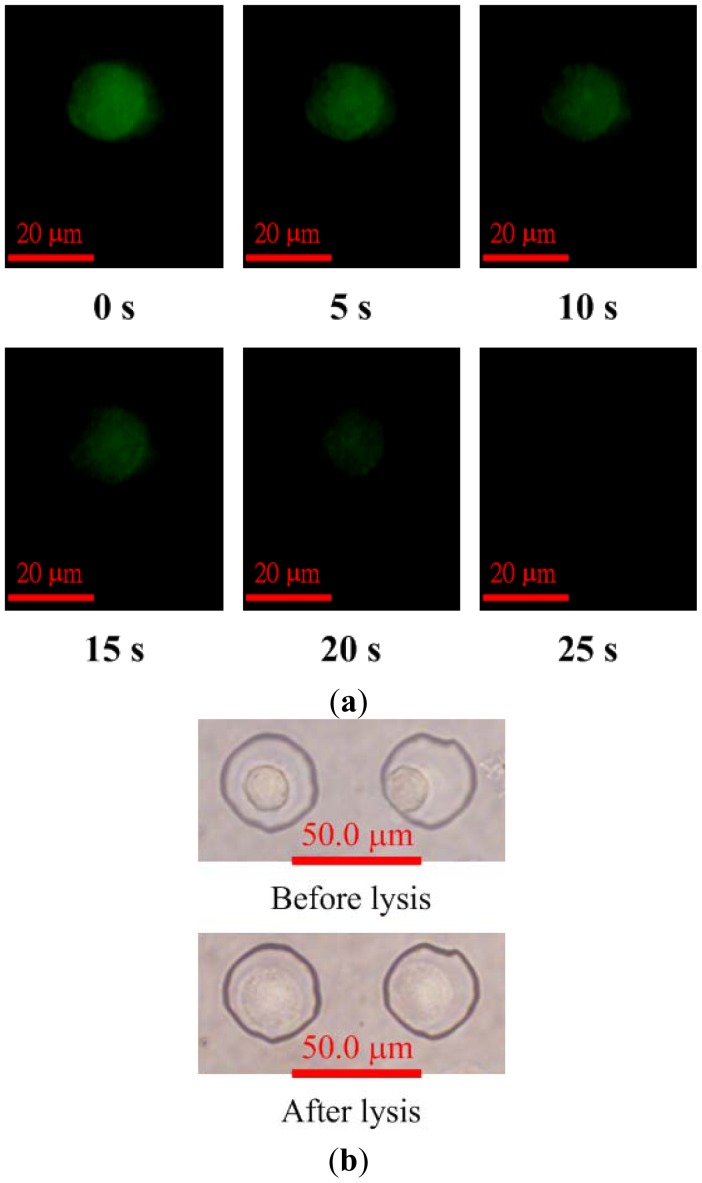
(**a**) Fluorescence images after applying the voltage of 30 V for electric lysis; (**b**) Bright-field images of a single HeLa cell before and after lysis.

Images showing fluorescence intensity of calcein AM-loaded HeLa cells after applying the voltage of 30 V for electric lysis are shown in Figure 6(a). The averaged electric field, which is the applied voltage divided by the distance between the electrodes, was about 2 kV/m and assumed to be unaffected by the presence of cells. The fluorescence images indicate that the cell membranes were gradually lysed as the voltage for electric lysis was applied. Calcein leakage occurs when cell membranes are damaged; therefore, the fluorescence intensity within the cell decreases after the cell starts to lyse. The cell was fully lysed after 25 s, as shown in Figure 6(a). The bright-field images of the cell before and after lysis are shown in Figure 6(b). The cell membrane was damaged by the electric field and the cell swelled. However, the cells remained in the microwells after lysis.

**Figure 7.**
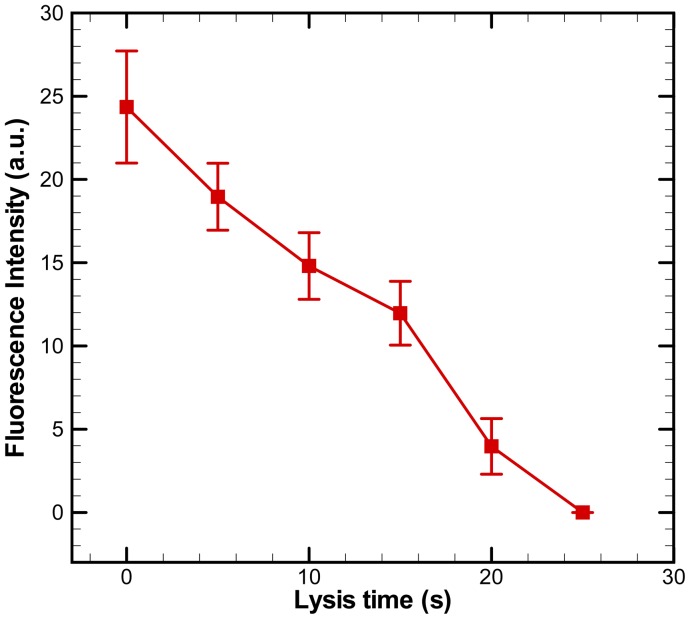
Fluorescence intensity of a single HeLa cell versus time after applying the voltage of 30 V for electric lysis. The experimental data are based on measurements of fluorescence in at least three individual cells. Each experimental data point represents the average value and the error bar shows the standard error of the mean.

The fluorescence intensities within single cells were quantified using ImageJ software. The fluorescence intensity of cells versus time is plotted in Figure 7. The experimental data are based on measurements of calcein intensity in at least three individual cells. Each experimental data point represents the average value, and the error bar shows the standard error of the mean. The intensity of calcein drops gradually during a DC voltage of 30 V for electric lysis was applied. The fluorescence intensity decreases to almost zero at 25 s after applying the voltage. Single-cell-based electric lysis in an electroosmotic-driven microfluidic device with microwells is thus feasible. The present approach of passively confining cells inside microwells is simple to implement and easy to integration. As a cell flows through a microchannel, it will gradually settle toward the bottom surface due to gravity and follow streamlines leading into the microwells while losing velocity. Therefore, the issue of stress-activated signaling pathways of cells in the microchips of the flow-based single cell positioning [28,29] could be avoided. The aforementioned approaches of patterning single cells in microwells for single cell analysis [15,22] required manual handling which was not reliable. The electroosmotic-driven microfluidic chip with microwells created by soft lithography are developed in this study, making it low-cost and easy to fabricate.

## 4. Conclusions

An electroosmotic-drive microfluidic chip with microwells were fabricated and investigated in the present work. The occupancy of cells in the 30-μm-diameter microwells was higher than 80% when the voltages of 5 and 10 V were applied. The occupancy of cells decreased with increasing the applied voltage. More than 50% of the occupied microwells contained individual cells. The results of electric lysis experiments at the single-cell level indicate that the cells were gradually lysed as the DC voltage of 30 V was applied; the cell was fully lysed after 25 s. The bright-field images of the cell before and after lysis indicate that the cell was lysed by the electric field. However, some cell lysate might dissipate in our microchips. The procedures of encapsulating cell lysate inside the microwells, for example, pressing the top PDMS sheet against the bottom one by microfluidic valves, should be investigated in the future. Moreover, the cell viability could be analyzed and remained during electroosmosis. The proposed microfluidic chips are suitable for high-throughput cell lysis and subsequent single-cell analysis, such as monitoring protein levels and enzymatic activities in a single cell, or single-cell PCR.
